# massiveGST: A Mann–Whitney–Wilcoxon Gene-Set Test Tool That Gives Meaning to Gene-Set Enrichment Analysis

**DOI:** 10.3390/e24050739

**Published:** 2022-05-23

**Authors:** Luigi Cerulo, Stefano Maria Pagnotta

**Affiliations:** 1 Department of Science and Technology, Università degli Studi del Sannio, 82100 Benevento, Italy; lcerulo@unisannio.it; 2 Bioinformatics Lab, Biogem, Molecular Biology and Genetics Research Institute, 83031 Ariano Irpino, Italy

**Keywords:** competitive enrichment methods, gene-profile, gene-sets ranking, Kolmogorov–Smirnov’s test, pathway analysis, rank sum test, Wilcoxon’s test

## Abstract

Gene-set enrichment analysis is the key methodology for obtaining biological information from transcriptomic space’s statistical result. Since its introduction, Gene-set Enrichment analysis methods have obtained more reliable results and a wider range of application. Great attention has been devoted to global tests, in contrast to competitive methods that have been largely ignored, although they appear more flexible because they are independent from the source of gene-profiles. We analyzed the properties of the Mann–Whitney–Wilcoxon test, a competitive method, and adapted its interpretation in the context of enrichment analysis by introducing a Normalized Enrichment Score that summarize two interpretations: a probability estimate and a location index. Two implementations are presented and compared with relevant literature methods: an R package and an online web tool. Both allow for obtaining tabular and graphical results with attention to reproducible research.

## 1. Introduction

Enrichment analysis (EA) of gene-sets is a technique typically used to uncover the phenotype of a gene-profile associated with the differential expression between two conditions [1] (e.g., treatment and control). If many genes (the gene-set) contribute to a phenotype or a cellular function, enrichment analysis tests whether a gene-set is associated with one of the two conditions [2]. The test procedures are classified as global or competitive tests [3]. In global test approaches, the test involves only genes in the gene-set. Instead, in competitive tests, the genes in the gene-set are compared with those outside the set. In this case, the test is applied to a gene-profile summarizing the differences between the two conditions. When ordered, from the highest to the lowest, a gene-profile is known as a pre-ranked list. An extensive and recent qualitative review of EA methods and tools is in [4].

To obtain the significance level, analytical methods are generally not applicable because the distributional hypothesis behind the test is not met. Computational strategies can help to estimate the null distribution by shuffling samples or genes. Since the seminal paper of [5], researchers mainly focused on shuffling samples leaving the inference from the gene-profile slightly covered. With [6], the analysis of a gene-profile becomes more central as EA was done at the level of the single sample profile.

GSEA [5] is the most adopted gene-set enrichment methodology. It is based on a modified version of the two-sample Kolmogorov–Smirnov (weighted-KS, wKS) test and is applied on a gene-profile. In this manuscript, GSEA and wKS are interchangeable. Basically GSEA consists of testing whether the distribution of scores associated with genes inside the gene-set is the same of the distribution of scores of genes outside the gene-set, i.e., $H_{0}$:$F_{\mathrm{in}}\left(x\right)=F_{\mathrm{out}}\left(x\right)$, toward the alternative $H_{1}$:$F_{\mathrm{in}}\left(x\right)\neq F_{\mathrm{out}}\left(x\right)$. Given the non-canonical form of the test-statistic, resampling methods help obtain the *p*-value. If the original data matrix that generated the gene-profile is available, samples are shuffled, the gene-profile is recomputed, and the test-statistic is evaluated. The empirical null distribution emerges repeating the shuffling several times. When the original data matrix is not available, starting from a pre-ranked lists of genes, the null distribution is computed by shuffling just gene names.

The hypothesis $H_{0}$:$F_{\mathrm{in}}\left(x\right)=F_{\mathrm{out}}\left(x\right)$ can be checked with the Mann–Whitney’s test-statistic [7] as well, and, with the help of Wilcoxon’s test-statistic [8], the computational effort of Mann–Whitney (MW) test decreases. In literature, the MW test is confused with Wilcoxon’s test or rank-sum test (RST). This overlap is misleading because Wilcoxon’s test is a test comparing the location of two populations, while MW’s test comparing the distribution functions is more general. To give relevance to the null hypothesis, we’ll refer to the MW test, supported by Wilcoxon, as MWW’s test. wKS and MWW share the same null hypothesis.

Both MWW and wKS tests have been proposed for EA. Table 1 summarizes the most relevant tools reported in literature.

A quantitative comparison of wKS and MWW EA algorithms, carried out by [15], states that the two methodologies are essentially equivalent in terms of significant gene-sets. A deeper study is in [16], where MWW and wKS are compared in the setting of weak functional signals, showing that MWW’s test is the most sensible.

In this work, we propose a new implementation of the enrichment analysis based on the MWW’s test (available as an easy-to-use web-based service and as an R package) called massiveGST (mGST). Current literature implementations essentially use the MWW’s test to compute the *p*-value associated with the gene-set. Instead, we exploit the statistical information from the test to obtain a richer view of the analysis. According to [17], the normalized version of the MW’s test-statistic is an estimate of probability. From such a probability, we propose two additional statistics, odds and logit2NES, that help researchers to understand the gene-set enrichment’s importance beyond the trivial evaluation of *p*-values. In addition, we propose: (1) a new prioritization of the tabular view of gene-sets EA that includes NES, *p*-value, and size of the gene-set; and (2) we demonstrate that the estimate of the probability owns a new interpretation as a location index. Then, our software provides a richer set of new statistics than available algorithms.

Furthermore, the computational effort to run the analysis has been compared with the EA tools reported in Table 1.

We ignored over-representation methodologies (e.g., [18]) based on the hypergeometric test, as they follow a completely different approach and include the theoretical issues of choosing the universe set and which genes are differentially expressed.

## 2. Materials and Methods

### 2.1. The Normalized Enrichment Score

The Normalized Enrichment Score and the *p*-value come from the Mann–Whitney’s test [7]. The null hypothesis $H_{0}$:$F_{\mathrm{in}}\left(x\right)=F_{\mathrm{out}}\left(x\right)$ states that there is no mutual dominance of the distribution functions, $F_{\mathrm{in}}\left(x\right)$ and $F_{\mathrm{out}}\left(x\right)$ that describe the intensities of genes, respectively, in and out of the gene-set. The alternative hypothesis states that the distribution function $F_{\mathrm{out}}\left(x\right)$ dominates $F_{\mathrm{in}}\left(x\right)$, i.e., $H_{1}$:$F_{\mathrm{out}}\left(x\right)>F_{\mathrm{in}}\left(x\right)$. Under the alternative hypothesis, the genes in the gene-set have intensities higher than those of the genes outside the gene-set. The MW test-statistic is:

$$U=\sum_{ij}I\left(x_{j}^{\mathrm{out}}<x_{i}^{\mathrm{in}}\right),$$

where I(·) is the indicator function. Basically, *U* is the number of times that the relation $x_{j}^{\mathrm{out}}<x_{i}^{\mathrm{in}}$ is true ∀i,j, where $x_{j}^{\mathrm{out}}$ ($j=1,2,\ldots ,m_{\mathrm{out}}$) is the intensity associated with the *j*th gene outside the gene-set, $x_{i}^{in}$ ($i=1,2,\ldots ,m_{\mathrm{in}}$) is the intensity associated with the *i*th gene in the gene-set, and $m=m_{\mathrm{in}}+m_{\mathrm{out}}$ is the total number of genes in the gene-profile. With the help of the Wilcoxon [8] test-statistic, the computation of *U* is drastically improved as follows:

$$U=m_{\mathrm{in}}\;m_{\mathrm{out}}+\frac{m_{\mathrm{out}}\left(m_{\mathrm{out}}+1\right)}{2}-T_{\mathrm{out}},$$

where $T_{\mathrm{out}}$ is the sum of rank transformed $x_{k}$, k=1,2,…,m outside the gene-set.

According to [17], the ratio $\frac{U}{m_{\mathrm{in}}\times m_{\mathrm{out}}}$ is an unbiased estimator of the probability $P\left[X_{\mathrm{in}}>X_{\mathrm{out}}\right]$, where $X_{\mathrm{in}}∼F_{\mathrm{in}}\left(x\right)$ and $X_{\mathrm{out}}∼F_{\mathrm{out}}\left(x\right)$. Given a gene-set, the event $X_{\mathrm{in}}>X_{\mathrm{out}}$ says that “*a gene randomly drawn from the gene-set has an intensity greater than the one of a second gene randomly sampled from outside the gene-set*”.

We define the estimate $\frac{U}{m_{\mathrm{in}}\times m_{\mathrm{out}}}$ of $P\left[X_{\mathrm{in}}>X_{\mathrm{out}}\right]$ as the Normalized Enrichment Score (NES) of a gene-set enrichment analysis. Assuming that a gene-profile recapitulates the differential expression of treatment samples versus control, an NES close to 1 means association of the gene-set with the treatment. Instead, an NES close to 0 suggests an association with the control group. This interpretation allows us to restate NES as

$$\mathrm{NES}=P\left[\mathrm{the}\mathrm{gene}-\mathrm{set}\mathrm{is}\mathrm{associated}\mathrm{with}\mathrm{the}\mathrm{treatment}\mathrm{group}\right]\approx \frac{U}{m_{in}\times m_{out}}.$$

A different way to look at the NES is the odds = NES/(1 − NES), i.e., the imbalance of the probability that the gene-set is associated with the treatment group to the probability that the gene-set has no association with it (or the gene-set is related to the control group).

$$\mathrm{odds}=\frac{P\left[\mathrm{the}\mathrm{gene}-\mathrm{set}\mathrm{is}\mathrm{associated}\mathrm{with}\mathrm{the}\mathrm{treatment}\mathrm{group}\right]}{P\left[\mathrm{the}\mathrm{gene}-\mathrm{set}\mathrm{is}\mathrm{not}\mathrm{associated}\mathrm{with}\mathrm{the}\mathrm{treatment}\mathrm{group}\right]}$$

The association with the treatment is as strong as the odds diverge to infinity; it is weak when the odds approach zero. In this last case, the association is with the control groups. An odds of about 1.0 means no association, neither the treatment nor the control.

A further transformation of NES is the

$$\mathrm{logit}2\mathrm{NES}=\log_{2}\left(\mathrm{odds}\right).$$

In this version of NES, a zero value means no association, a positive value means association with the treatment group, and a negative value means association with the control group.

The NES owns a descriptive interpretation as location index of the gene-set. It is the *percentile rank* of the gene-set, seen as a single value, in the ranking of the genes outside the gene-set (see Appendix A for the proof). When NES reaches 1, then genes in the gene-set are located at the top of the gene-profile. When NES is 0, the location is at the bottom and the association is with the control group.

### 2.2. Enrichments Prioritization

With the rapid growth of gene-sets collections, there is a problem of prioritizing significant results. In GSEA, gene-sets are generally ordered according to the NES or the *p*-value. However, this can be misleading because NES and gene-set size are dependent as shown by the following experiment.

**Figure 1:**
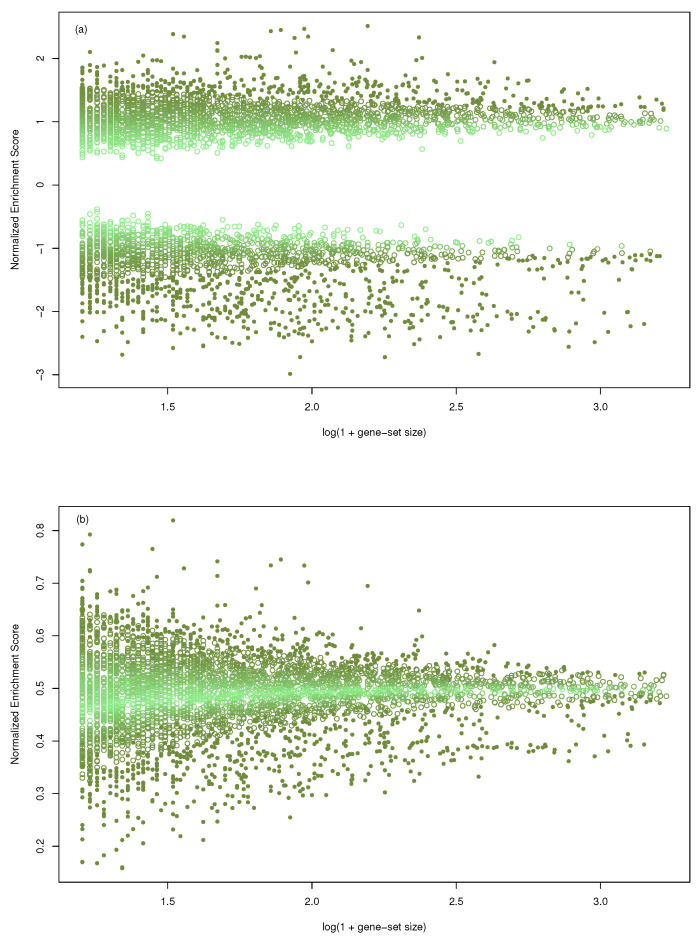
Scatter plot of the size of the gene-sets (transformed as $\log_{10}\left(1+\mathrm{size}\right)$) against the Normalized Enrichment Score; (**a**) in the case of GSEA, (**b**) for massiveGST. Data come from the gene-profile included in the R-package and 4046 gene-sets. The intensity of the color is proportional to the *p*-value (light color assigned to higher *p*-value).

We considered the gene-sets collection C5/BP from MSigDB [19] and the gene-profile published in [16]. Due to gene-set size, GSEA restricted the original collection to 4046 out of 7658. The same collection was used with mGST. In Figure 1, the size of the gene-sets (transformed as $\log_{10}\left(1+\mathrm{size}\right)$) has been plotted against the normalized enrichment score, both for GSEA (a) and mGST (b). The range of NES decreases as the size increases in both cases, showing a dependence. Furthermore, we measured the intensity of the dependence with the mutual information (computed with k-NN estimator implemented by [20]) obtaining $MI_{\mathrm{GSEA}}=0.0446$, and $MI_{\mathrm{mGST}}=0.0902$.

$MI_{\mathrm{GSEA}}=0.0446$, and $MI_{\mathrm{mGST}}=0.0902$ showing that exists dependence.

To improve the gene-sets prioritization and give more evidence to large ones, we propose an additional gene-sets score, named *relevance*, that aggregates NES, *p*-value, and gene-set size.

Let us assume that we run a two-sided enrichment test so that some gene-sets have logit2NES ≥0, and some others logit2NES < 0. For the $k^{\prime }$th gene-set, $k^{\prime }=1,2,\ldots$, in the collection having logit2NES ≥0, then

$${\mathrm{relevance}}_{k^{\prime }}^{+}=\mathrm{rank}\left({\mathrm{actual}-\mathrm{size}}_{k^{\prime }}\right)+\mathrm{rank}\left({\mathrm{logit}2\mathrm{NES}}_{k^{\prime }}\right)+\mathrm{rank}\left(1-p{-\mathrm{value}}_{k^{\prime }}\right),$$

where $\mathrm{rank}\left(\cdot \right)$ is a function that associates the highest rank with the highest value of its argument, and actual-size is the gene-set size. Similarly, the relevance in the subsets of gene-sets (with index $k^{\prime \prime }$) such that logit2NES<0 is

$${\mathrm{relevance}}_{k^{\prime \prime }}^{-}=\mathrm{rank}\left({\mathrm{actual}-\mathrm{size}}_{k^{\prime \prime }}\right)+\mathrm{rank}\left(-{\mathrm{logit}2\mathrm{NES}}_{k^{\prime \prime }}\right)+\mathrm{rank}\left(1-p{-\mathrm{value}}_{k^{\prime \prime }}\right).$$

Finally, given the *k*th gene-set,

$${\mathrm{relevance}}_{k}=\left\{\begin{array}{c} {\mathrm{relevance}}_{k}^{+}\Leftrightarrow \mathrm{logit}2\mathrm{NE}S_{k}\geq 0\\ {\mathrm{relevance}}_{k}^{-}\Leftrightarrow \mathrm{logit}2\mathrm{NE}S_{k}<0 \end{array}\right.$$

In the case of “greater” (less) alternative hypothesis, relevance${}_{k}\equiv$ relevance${}_{k}^{+}$ (relevance${}_{k}\equiv$ relevance${}_{k}^{-}$).

### 2.3. Enrichments Visualization

We integrated the tabular results with a network-graph of gene-sets. A node represents a significant gene-set. The size of node is proportional to the size of gene-sets, while the intensity of the color is proportional to NES values. The connection between two gene-sets *A* and *B* is proportional to their similarity S(A,B). The similarity S(A,B) is computed as a convex combination of the Jaccard, $\delta_{0}\left(A,B\right)=\left|A\cap B|/|A\cup B\right|$, and the overlap, $\delta_{1}\left(A,B\right)=\left|A\cap B\right|/\min\left(|A|,|B|\right)$, indexes.

$$S\left(A,B\right)=\epsilon \times \delta_{1}\left(A,B\right)+\left(1-\epsilon \right)\times \delta_{0}\left(A,B\right),$$

with 0≤ϵ≤1. When ϵ=0, we obtain $S\left(A,B\right)\equiv \delta_{0}\left(A,B\right)$, while ϵ=1 means $S\left(A,B\right)\equiv \delta_{1}\left(A,B\right)$.

### 2.4. Web-Based Service

**Figure 2:**
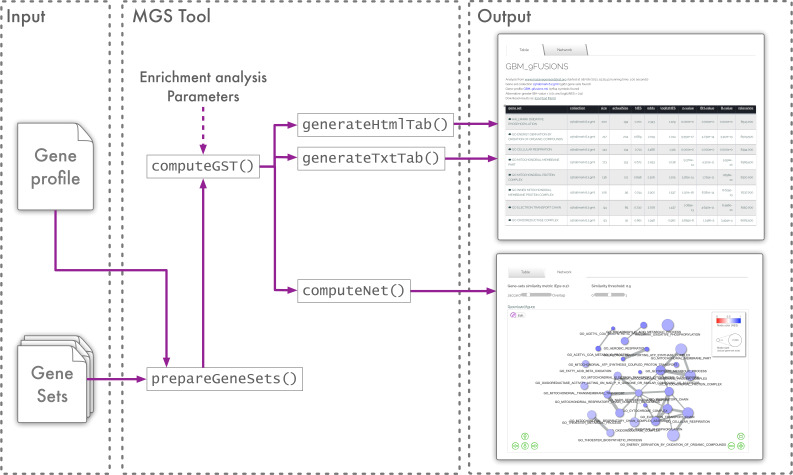
Software architecture of the online web-service.

A simplified functional architecture of the mGST Tool is shown in Figure 2. It is implemented in Javascript and is executed on the client host. Gene-set pre-elaboration is performed by the prepareGeneSets() function. Basically, it computes gene-profile ranking in O(m×log(m)) time, where *m* is the length of the gene-profile, and collects global information in appropriate data structures, such as the total number of genes and the sum of ranks.

The core of the algorithm is implemented in the computeGST() function, where, for each gene-set, ranking and test-statistics are computed in linear time. Results are collected in an interactive html table and can be exported in csv, tsv, and html formats. The computeNet() function performs additional network analysis and generates a graph representation of the results that can be exported in png format. User interface interaction features are implemented by using html5 and ajax frameworks.

### 2.5. R Package

The R package is a collection of functions to compute the enrichment analysis and to manipulate and plot the results. The primary function is massiveGST that needs as mandatory input the gene-profile and the collection of the gene-sets. The output is a data frame arranging all the statistics introduced in the methodology section. Three functions cut_by_NES, cut_by_logit2NES, and cut_by_significance trim the data frame according to the required constraints. With the help of the S3-method, the function plot provides a graphical display for the analysis. The enrichments can be presented as a bar plot or as a network.

**Figure 3:**
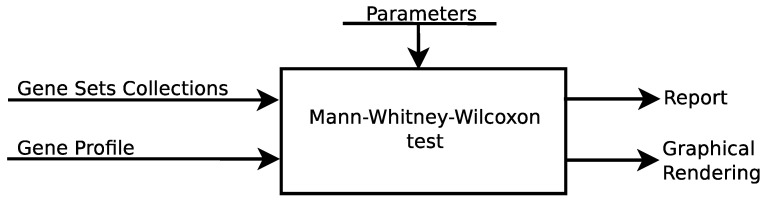
Flow-chart to run analysis both in the web service, and in the R environment.

The logical scheme is shown in Figure 3. An extensive presentation of the package usability is in the vignette at https://cran.r-project.org/web/packages/massiveGST/vignettes/vignette.html (accessed on 11 April 2022).

## 3. Results

### 3.1. Computational Time: Comparison with Literature Methods

To assess the computational efficiency of our proposal, we designed a simulation experiment involving real data from TCGA. With the help of TCGAbiolinks [21], we downloaded data and annotations from different studies. We got gene-profiles by comparing subtypes by using a DESeq2 package [22]. The gene-profile is $-log\left(p_{j}\right)\times \mathrm{sign}\left(W_{j}\right)$, across genes, where $p_{j}$ and $W_{j}$ are the *p*-value and the test-statistic of the Wald’s test, respectively. In total, we collected 30 gene-profiles.

We screened nine recent literature proposals for enrichment analysis both as R-package and online service shown in Table 1.

**Figure 4:**
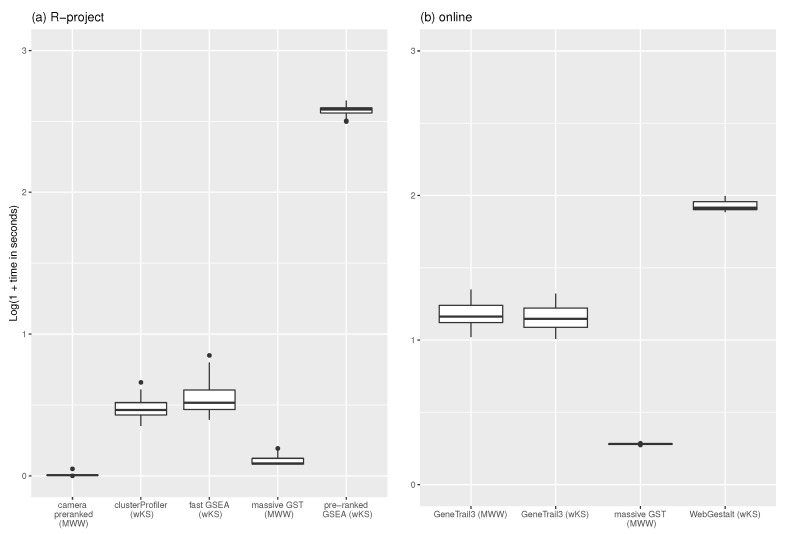
Results of the simulation. 30 gene-profiles have been queried with MSigDB C1 collection of 278 gene-sets using R-implementation of the methodologies (**a**): clusterProfiler with DOSE and fGSEA options, fast GSEA, pre ranked GSEA, massive GST, and camera pre-ranked) and online tools (**b**): GeneTrial3 with weighted GSEA and Wilcoxon Rank Sum test options, massive GST, and WebGestalt GSEA). The time, in seconds, is $\log_{10}$ transformed. The raw data are in Table A1.

The 30 gene-profiles, together with the C1 collection of 278 positional gene-sets from MSigDB [19], fed the nine procedures. Table A1 shows the computational time (in seconds) measured on a PC running Ubuntu with Kernel Linux 5.4.0-73-generic x86_64 (4 cores, 16 GB RAM), and Google Chrome Version 90.0.4430.212 (64-bit). Figure 4 shows a boxplot of the experiment results. The time has been transformed as $\log_{10}\left(1+\mathrm{time}\right)$ to bound the different ranges of each procedures. Camera pre-ranked (on average 0.02 s with 0.03 as standard deviation) and massive GST (0.27 s with 0.10 as standard deviation) own the lowest computation time in the R environment, confirming results reported in [23]. The time difference between massive GST and camera pre-ranked is because the latter applies the MWW’s test and returns the *p*-value with an indicator of the direction of the test; instead, massive GST provides the statistics presented in the methodology section. As online service, our proposal spends 0.91 s on average (sd = 0.01), versus 13.57 (sd = 3.01) of wKS (GeneTrail3) and 14.30 (sd = 3.13) of MWW (GeneTrail3). WebGestalt (wKS) spends 84.20 s (sd = 6.69).

### 3.2. Usage of the Online Web-Tool

To run the analysis, the user needs to load two files: (a) a gene-profile (as a two columns tab-separated text format, the gene-name and the associated value), and (b) one or more gene-sets collections (in .gmt format).

The next steps are: (1) set the significance-level of the enrichments (the user can choose between the *p*-value, and two versions of adjusted *p*-values: Benjamini–Hochberk and Bonferroni), and (2) (optionally) set the threshold value of the logit2NES.

From the user’s point of view, the online web tool follows the same logical scheme as Figure 3.

The significance level allows for selecting gene-sets relevant for the treatment and control. In addition, the researcher could be interested in those gene-sets strongly associated. In this case, the trimming with NES, both as location index or probability, comes into play. NES could be difficult to handle and read because it is a positive number, and people have to remember that association with treatment or controls depends on the value above or below 0.5, respectively. As a help, the logit2NES simplifies the process of interpreting the association (positive values with the treatment, negative with the control) and intuitively measuring the strongness of association (higher positive values mean strong association; lower negative values signify strong association with the control). The equivalence of values from NES, odds, and logit2NES is shown in Table 2.

To require that the probability of association of the gene-set with the treatment group be about twice the probability of non association, the logit2NES threshold can be set to 0.9 (equivalent to NES > 0.65, or odds > 1.86).

**Figure 5:**
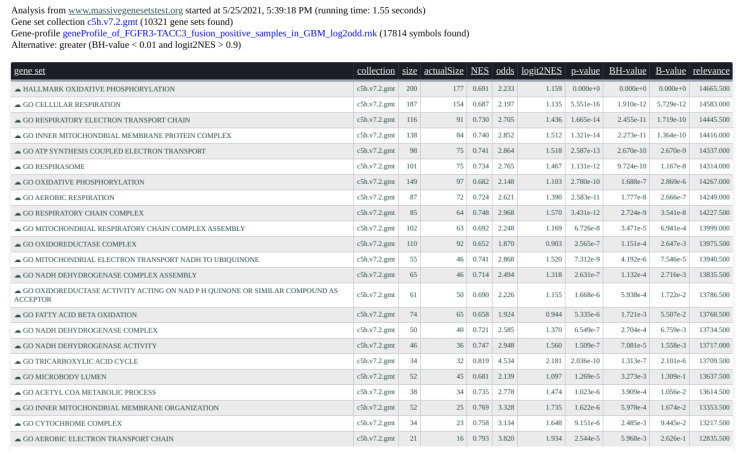
Screenshot of the tabular results of the gene-profile associated with FGFR3-TACC3 fusion positive samples in GBM. C5 and Hallmark collections (in total 10,321 gene-sets) from MSigDB interrogated the gene-profile in 1.55 s.

Tabular versions of results are also generated (see Figure 5). The shown report respects the constrains given as input, while the full table with every gene-set can be downloaded as .csv or .tsv formats. The html version of the table can be downloaded as shown. Both the displayed table and its .html version allow the user to re-sort results according to any column.

To visualize the network-graph of current results, the user can click on the network tab. Here, the similarity between any two of the gene-sets in the table is computed and the network of gene-sets is shown. The user can chose between two similarity measures, Jaccard or overlap, or any convex combination of the twos by tuning the parameter ϵ with a slider box. A second slider-box allows for setting the threshold value so that a segment joins two nodes when the similarity is above it. The network is updated in real time, as the user operates with the sliders. The plot of the network allows some editing actions and it can be downloaded as a .png file.

The page http://www.massivegenesetstest.org/gettingStarted.html (accessed on 11 April 2022) from the web-site helps to run a first example analysis.

**Figure 6:**
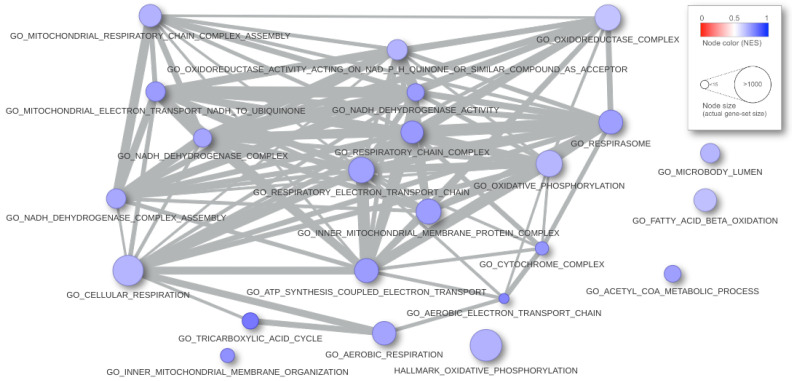
Graphical rendering of the tabular results of the analysis. Each ball is a gene-set; the radius matches the dimension, and the color corresponds to the NES. When two gene-sets share some gene, they appear connected, and the strength of similarity results in the thickness of the segment.

In Figure 5, we present an example of result report. We interrogated the gene-profile of the *FGFR-TACC3 fusion positive samples in the glioblastoma multiforme* study from the TCGA (see [16]) with the C5 and Hallmark collections (MsigDB v.7.2) of 10,321 gene-sets from the Broad Institute. The computation time took 1.55 s. The input parameters are alternative = greater, B.value < 0.01, and abs(logit2NES) > 1. In Figure 6, the graphical rendering of the significant gene-sets is shown.

## 4. Conclusions, Limitations, and Future Research

Gene-set enrichment analysis is a methodology of great interest in silico experiments. Its first aim is to give a biological meaning associated with genes profiles coming as result of any analysis. Since its introduction, effort has been spent to improve the results’ reliability and extend the field of application. Much attention has been devoted to global test versions, but competitive methods, requiring just a gene-profile, appear more flexible because the profile can be generated with up-to-date methodology (the case of analyzing a single cell is an example).

GSEA is the most adopted methodology, with about 32,000 citations to date. A similar approach is offered by competitive tests involving the Mann–Whitney–Wilcoxon test. To date, such a test is offered as an optional alternative in several other methodologies, but the theoretical properties have not been exploited.

In this paper, we have presented the massiveGST procedure, implemented as an R package and available as a web-tool, centered on MWW’s test methodology for competitive gene-set enrichment analysis. We exploited the theoretical knowledge of the test to improve the interpretation of the enrichment results. We proposed the interpretation of the normalized version of MWW’s test-statistic as an estimate of probability and as a location index in the ordered universe of genes outside the gene-set. Convincing use of this last interpretation is in [24].

As demonstrated in the simulation experiment, enrichment analysis with MWW’s test generally requires low computation time. In the R environment, the massiveGST function competes with cameraPR but offers a rich set of statistics. Our online implementation is the most competitive (about 1.5 s for more than 10,000 gene-sets).

A general issue is the lack of an independent paradigm to test which method/procedure is reliable. Something has been done with the recent contribution from [23], where real datasets of pathologies have been selected and, for each of them, genes associated with the pathology have been gathered from the literature. The gene-sets containing such genes have been assumed as ground truth. The assumption is that a gene associated with a pathology should be highly differentially expressed with respect to control samples. Such a hypothesis neglects that a large subset of weak or moderate signal genes cooperates with important biological phenotypes [25], posing critical concerns on the usage of the paradigm proposed in [23] for the evaluation of EA methods.

Competitive EA methods have increased attention in applied research as they own implicit adaptability to emerging new omic technologies. It is urgent to design a comparison paradigm with large consensus to know the strengths and weaknesses of methodologies, such as those developed in other contexts (e.g., DREAM, KAGGLE, …[26]).

The availability of a fast methodology for EA, together and results not affected by variability induced by the computational strategy to obtain the significance, could push new contributions to methodological proposals in discovering master regulators (e.g., [27]). Such tools, starting from the estimation of gene regulatory network [28,29], apply EA methods to detect those transcription factors able to drive phenotypes.

Competitive EA methods may have several applications in fields also far from bioinformatics. Consider a list of items (think about the ranking of basketball players), sorted according to some criterium (the best player at the top), and different ways to cluster them (the teams, the ethnicity, young players, …). The result of the competitive EA method with MWW will be the location in the ranked list of a consistent cluster of items (the young basketball players perform better than others, in the case that the cluster is located close to the top).

## Figures and Tables

**Table 1:** State-of-the-art implementation of enrichment analysis tools based on the wKS and MWW test statistics.

EA Tool	Reference	Year	Test	Available as
camera	[2]	2012	MWW	R function in limma package
GSEA	[5]	2005	wKS	R package
fGSEA	[9]	2021	wKS	R package
clusterProfiler	[10]	2012	wKS	R package
massiveGST	[11,12]	2022	MWW	R package/web
GeneTrial3	[13]	2020	wKS/MWW	web
WebGestalt	[14]	2019	wKS	web

**Table 2:** Table of equivalence among NES, odds, and logit2NES.

NES	Odds	logit2NES
0.20	0.25	−2.00
0.30	0.43	−1.22
0.40	0.67	−0.58
0.50	1.00	0.00
0.60	1.50	0.58
0.65	1.86	0.90
0.75	3.00	1.58
0.90	9.00	3.17

## Data Availability

The 30 gene-profiles generated for the simulation can be retrieved from http://www.massivegenesetstest.org/gene_profiles/ (accessed on 11 April 2022).

## Software Availability

The online service is at http://www.massivegenesetstest.org/ (accessed on 11 April 2022) and requires Safari, Chrome, Firefox and InternetExplorer. The R package can be downloaded from https://CRAN.R-project.org/package=massiveGST (accessed on 11 April 2022) (also at https://github.com/stefanoMP/massiveGST (accessed on 11 April 2022)) and requires at least the v. 4.1.0 of the R environment.

## Abbreviations

The following abbreviations are used in this manuscript:

GSEA	Gene-Set Enrichment Analysis
EA	Enrichment Analysis
ES	Enrichment Score
NES	Normalized Enrichment Score
GST	Gene-Set Test
mGST	massive Gene-Set Test
MWW	Mann–Whitney–Wilcoxon
RST	Rank-Sum test
KS	Kolmogorov–Smirnov
wKS	weighted-KS
MI	Mutual Information
TCGA	The Cancer Genome Atlas
k-NN	k-Nearest Neighbor

## Appendix A

**Theorem** **A1.**
*The Normalized Enrichment Score is a location index.*

**Proof.** Let $T_{\mathrm{in}}$ be the sum of the $m_{\mathrm{in}}$ ranks inside the gene-set, while $T_{\mathrm{out}}$ is the sum of the $m_{\mathrm{out}}$ outside, then

$$T_{\mathrm{in}}+T_{\mathrm{out}}\equiv \frac{m(m+1)}{2},$$

where $m=m_{\mathrm{in}}+m_{\mathrm{out}}$. If we set $U_{\mathrm{in}}=T_{\mathrm{in}}-m_{\mathrm{in}}\left(m_{\mathrm{in}}+1\right)/2$ and $U_{\mathrm{out}}=T_{\mathrm{out}}-m_{\mathrm{out}}\left(m_{\mathrm{out}}+1\right)/2$, it can be shown that

$$U_{\mathrm{in}}+U_{\mathrm{out}}=m_{\mathrm{in}}\times m_{\mathrm{out}},$$

in fact

$$U_{\mathrm{in}}+U_{\mathrm{out}}=T_{\mathrm{in}}-m_{in}\left(m_{\mathrm{in}}+1\right)/2+T_{\mathrm{out}}-m_{\mathrm{out}}\left(m_{\mathrm{out}}+1\right)/2=$$

$$=\frac{m(m+1)}{2}-\frac{m_{\mathrm{in}}\left(m_{\mathrm{in}}+1\right)+m_{\mathrm{out}}\left(m_{\mathrm{out}}+1\right)}{2}$$

$$=\frac{m(m+1)}{2}-\frac{m_{\mathrm{in}}^{2}+m_{\mathrm{in}}+m_{\mathrm{out}}^{2}+m_{\mathrm{out}}\pm 2m_{\mathrm{in}}m_{\mathrm{out}}}{2}=$$

$$=\frac{m(m+1)}{2}-\frac{{\left(m_{\mathrm{in}}+m_{\mathrm{out}}\right)}^{2}+\left(m_{\mathrm{in}}+m_{out}\right)-2m_{\mathrm{in}}m_{\mathrm{out}}}{2}=$$

$$=\frac{m(m+1)}{2}-\frac{m^{2}+m-2m_{\mathrm{in}}m_{\mathrm{out}}}{2}=m_{\mathrm{in}}m_{\mathrm{out}}.$$

From these relations, we obtain that MW’s *U* statistic corresponds to $U_{\mathrm{in}}$, and

$$0\leq \frac{U}{m_{\mathrm{in}}\times m_{\mathrm{out}}}\leq 1.$$

Our interest is in

$$0\leq \frac{U}{m_{\mathrm{in}}}\leq m_{\mathrm{out}}.$$

We can show that $U/m_{\mathrm{in}}\equiv m_{\mathrm{out}}$, when $T_{\mathrm{in}}$ sums the highest *k* ranks. In this case, $m_{\mathrm{in}}=k$, and $m_{\mathrm{out}}=m-k$;

$$T_{\mathrm{in}}=m+\left(m-1\right)+\left(m-2\right)+\cdots +\left(m-k+1\right)=k\times m-\frac{k(k-1)}{2}$$

$$U=T_{\mathrm{in}}-\frac{k(k+1)}{2}=k\times m-\frac{k(k-1)}{2}-\frac{k(k+1)}{2}=k\times m-k^{2}$$

$$\frac{U}{k}=\frac{k\times m-k^{2}}{k}=m-k\equiv m_{\mathrm{out}}.$$

Conversely, when $T_{\mathrm{in}}$ sums the lowest *k* ranks, then $T_{\mathrm{in}}=\frac{k(k+1)}{2}$, and $\frac{U}{k}\equiv 0$. The ratio $\frac{U}{m_{\mathrm{in}}\times m_{\mathrm{out}}}$ is the *percentile rank* of the gene-set, seen as a single value, in the ranking of the genes outside the gene-set. □

**Table A1:** Results of the simulation ${}^{1}$. Thirty gene-profiles were queried with MSigDB C1 collection (V.7.4) of 278 gene-sets. Six procedures come from R implementation GSEA, fastGSEA (fGSEA), clusterProfiler (CP) with fGSEA option, massiveGST (mGST), and camera pre-ranked (cPR); four more results come from online services GeneTrial3 (weighted GSEA and Wilcoxon Rank Sum test), massiveGST, GSEA offered by WebGestalt. The values are in seconds. The last two rows report the average and the standard deviation across the 30 experiments. The study corresponds to the name of the cancer collection of samples in TCGA; DOI maps to the paper from which the sub-typing of samples was obtained; control is the subtype assumed as the control group, while treatment is a second subtype. In brackets, there is the number of samples in the groups. The length refers to the number of genes in the gene-profile. File name is the file-name of the gene-profile.

					R Project					Online				
Study	Doi	Control	Treatment	Length	GSEA	fGSEA	CP/wKS	mGST	cPR	mGST	GT3/MWW	GT3/wKS	WG/wKS	File Name ^2^
BLCA	10.1016/j.cell.2017.09.007	Basal-squamous (142)	Luminal (246)	19,664	319.59	2.09	1.79	0.21	0.01	0.93	19.97	16.78	90.26	BLCA_Wald_pv.rnk
BRCA	10.1016/j.ccell.2018.03.014	Basal (190)	Her2 (82)	19,579	314.71	1.85	1.79	0.22	0.01	0.91	12.92	13.45	78.38	BRCA_BH2_Wald_pv.rnk
BRCA	10.1016/j.ccell.2018.03.014	Basal (190)	LumA (562)	19,657	317.99	1.92	2.04	0.23	0.01	0.91	13.15	13.07	80.55	BRCA_BLA_Wald_pv.rnk
BRCA	10.1016/j.ccell.2018.03.014	Basal (190)	LumB (209)	19,626	321.35	2.04	1.99	0.21	0.01	0.89	12.89	13.88	77.99	BRCA_BLB_Wald_pv.rnk
BRCA	10.1016/j.ccell.2018.03.014	Her2 (82)	LumA (562)	19,650	383.10	2.08	1.87	0.23	0.01	0.91	13.17	14.21	86.20	BRCA_H2LA_Wald_pv.rnk
BRCA	10.1016/j.ccell.2018.03.014	Her2 (82)	LumB (209)	19,592	380.24	2.01	1.85	0.21	0.01	0.91	13.95	15.17	75.52	BRCA_H2LB_Wald_pv.rnk
BRCA	10.1016/j.ccell.2018.03.014	LumA (562)	LumB (209)	19,652	380.88	2.59	1.93	0.36	0.01	0.92	15.83	16.92	95.24	BRCA_LALB_Wald_pv.rnk
KIRC	10.1038/nature12222	1 (147)	2 (90)	19,639	393.28	6.07	3.56	0.21	0.01	0.91	18.02	21.32	77.28	KIRC_1_2_Wald_pv.rnk
KIRC	10.1038/nature12222	1 (147)	3 (94)	19,609	376.26	2.88	1.90	0.37	0.01	0.91	18.09	17.42	76.29	KIRC_1_3_Wald_pv.rnk
KIRC	10.1038/nature12222	1 (147)	4 (86)	19,613	407.56	3.55	2.83	0.38	0.01	0.91	16.54	18.38	98.32	KIRC_1_4_Wald_pv.rnk
KIRC	10.1038/nature12222	2 (90)	3 (94)	19,633	401.31	3.50	2.57	0.38	0.01	0.91	17.44	20.10	81.18	KIRC_2_3_Wald_pv.rnk
KIRC	10.1038/nature12222	2 (90)	4 (86)	19,638	382.03	1.86	1.38	0.21	0.01	0.92	17.76	20.13	81.32	KIRC_2_4_Wald_pv.rnk
KIRC	10.1038/nature12222	3 (94)	4 (86)	19,609	389.74	3.28	2.21	0.40	0.01	0.91	18.32	19.36	80.15	KIRC_3_4_Wald_pv.rnk
LGG	10.1016/j.cell.2015.12.028	IDHwt (97)	IDHmut (419)	19,661	387.23	2.92	2.31	0.23	0.01	0.91	15.16	15.19	79.88	LGG_IDH_Wald_pv.rnk
LUAD	10.1038/nature13385	inflammatory (141)	proliferative (89)	19,542	383.93	2.45	2.08	0.43	0.01	0.90	9.63	10.79	95.65	LUAD_InflamProl_Wald_pv.rnk
LUAD	10.1038/nature13385	proximal (78)	TRU (63)	19,469	376.99	2.05	1.78	0.22	0.01	0.90	9.76	10.10	82.60	LUAD_ProxTRU_Wald_pv.rnk
LUSC	10.1038/nature11404	basal (43)	classical (65)	19,560	383.54	4.31	2.67	0.21	0.01	0.91	9.54	10.65	92.81	LUSC_BC_Wald_pv.rnk
LUSC	10.1038/nature11404	basal (43)	primitive (27)	19,554	378.42	2.85	2.05	0.22	0.01	0.90	9.90	12.12	82.13	LUSC_BP_Wald_pv.rnk
LUSC	10.1038/nature11404	basal (43)	secretory (44)	19,554	389.57	3.06	2.56	0.24	0.01	0.90	10.36	10.93	88.52	LUSC_BS_Wald_pv.rnk
LUSC	10.1038/nature11404	classical (65)	secretory (44)	19,481	421.33	5.30	3.07	0.21	0.01	0.90	11.99	13.16	80.77	LUSC_CS_Wald_pv.rnk
LUSC	10.1038/nature11404	primitive (27)	secretory (44)	19,481	411.08	5.09	3.02	0.21	0.12	0.89	11.32	12.44	97.50	LUSC_PS_Wald_pv.rnk
PAAD	10.1016/j.ccell.2017.07.007	classical (54)	exocrine (62)	19,395	381.77	1.75	1.46	0.20	0.01	0.89	13.04	12.93	76.34	PAAD_classical_exocrine_Wald_pv.rnk
PAAD	10.1016/j.ccell.2017.07.007	classical (54)	QM (34)	19,334	393.19	1.97	1.56	0.50	0.01	0.88	9.18	9.48	78.63	PAAD_classical_QM_Wald_pv.rnk
PAAD	10.1016/j.ccell.2017.07.007	exocrine (62)	QM (34)	19,366	412.21	1.49	1.25	0.21	0.01	0.88	10.27	11.63	80.78	PAAD_exocrine_QM_Wald_pv.rnk
STAD	10.1038/nature13480	C1 (49)	C2 (59)	19,648	442.49	1.73	1.66	0.22	0.01	0.91	12.84	11.81	85.34	STAD_C1C2_Wald_pv.rnk
STAD	10.1038/nature13480	C1 (49)	C3 (98)	19,679	442.85	1.83	1.59	0.22	0.12	0.91	11.21	12.46	91.14	STAD_C1C3_Wald_pv.rnk
STAD	10.1038/nature13480	C1 (49)	C4 (48)	19,651	351.94	1.56	1.41	0.22	0.00	0.91	12.46	13.18	90.15	STAD_C1C4_Wald_pv.rnk
STAD	10.1038/nature13480	C2 (59)	C3 (98)	19,681	357.27	2.38	1.62	0.56	0.01	0.91	14.10	13.64	78.59	STAD_C2C3_Wald_pv.rnk
STAD	10.1038/nature13480	C2 (59)	C4 (48)	19,664	339.91	2.52	1.83	0.21	0.01	0.91	13.01	14.27	86.03	STAD_C2C4_Wald_pv.rnk
STAD	10.1038/nature13480	C3 (98)	C4 (48)	19,681	344.40	2.17	2.05	0.22	0.12	0.91	15.15	14.13	80.49	STAD_C3C4_Wald_pv.rnk
average					378.87	2.70	2.06	0.27	0.02	0.91	13.57	14.30	84.20	
standard deviation					33.05	1.14	0.54	0.10	0.03	0.01	3.01	3.13	6.69	

^1^ The experiments have run on a PC equipped with Intel(c) Xeon(R) CPU E3-1226 v3 @ 3.30 GHz × 4 cores and 16 GB RAM. The OS is the Linux Mint v. 20.3 with Kernel Linux 5.4.0-73-generic x86_64. The R experiments have run in the R v. 4.1.3 environment, while the online web experiments have run in the Google Chrome v. 90.0.4430.212 (64-bit) browser. ^2^ The files are available at http://www.massivegenesetstest.org/gene_profiles/ (accessed on 11 April 2022).

## References

[1] Mootha V., Lindgren C., Eriksson K., Subramanian A., Sihag S., Lehar J., Puigserver P., Carlsson E., Ridderstråle M., Laurila E. (2003). PGC1*α*-responsive genes involved in oxidative phosphorylation are coordinately downregulated in human diabetes. Nat. Genet..

[2] Wu D., Smyth G. (2012). Camera: A competitive gene set test accounting for inter-gene correlation. Nucleic Acids Res..

[3] Tian L., Greenberg S., Kong S., Altschuler J., Kohane I., Park P. (2005). Discovering statistically significant pathways in expression profiling studies. Proc. Natl. Acad. Sci. USA.

[4] Das S., McClain C.J., Rai S.N. (2020). Fifteen Years of Gene Set Analysis for High-Throughput Genomic Data: A Review of Statistical Approaches and Future Challenges. Entropy.

[5] Subramanian A., Tamayo P., Mootha V., Mukherjee S., Ebert B., Gillette M., Paulovich A., Pomeroy S., Golub T., Lander E. (2005). Gene set enrichment analysis: A knowledge-based approach for interpreting genome-wide expression profiles. Proc. Natl. Acad. Sci. USA.

[6] Barbie D.A., Tamayo P., Boehm J.S., Kim S.Y., Moody S.E., Dunn I.F., Schinzel A.C., Sandy P., Meylan E., Scholl C. (2009). Systematic RNA interference reveals that oncogenic KRAS-driven cancers require TBK1. Nature.

[7] Mann H., Whitney D. (1947). On a Test of Whether one of Two Random Variables is Stochastically Larger than the Other. Ann. Math. Statist..

[8] Wilcoxon F. (1945). Individual Comparisons by Ranking Methods. Biom. Bull..

[9] Korotkevich G., Sukhov V., Budin N., Shpak B., Artyomov M.N., Sergushichev A. (2021). Fast gene set enrichment analysis. bioRxiv.

[10] Yu G., Wang L.G., Han Y., He Q.Y. (2012). clusterProfiler: An R Package for Comparing Biological Themes Among Gene Clusters. OMICS.

[11] Pagnotta S.M. (2022). massiveGST: Competitive Gene Sets Test with the Mann–Whitney–Wilcoxon Test. R Package Version 1.0.0. https://CRAN.R-project.org/package=massiveGST.

[12] Cerulo L., Pagnotta S.M. (2019). Massive Gene-Sets Test. http://www.massiveGeneSetsTest.org.

[13] Gerstner N., Kehl T., Lenhof K., Müller A., Mayer C., Eckhart L., Grammes N.L., Diener C., Hart M., Hahn O. (2020). GeneTrail 3: Advanced high-throughput enrichment analysis. Nucleic Acids Res..

[14] Liao Y., Wang J., Jaehnig E.J., Shi Z., Zhang B. (2019). WebGestalt 2019: Gene set analysis toolkit with revamped UIs and APIs. Nucleic Acids Res..

[15] Stöckel D., Kehl T., Trampert P., Schneider L., Backes C., Ludwig N., Gerasch A., Kaufmann M., Gessler M., Graf N. (2016). Multi-omics enrichment analysis using the GeneTrail2 web service. Bioinformatics.

[16] Frattini V., Pagnotta S., Fan J., Russo M., Lee S., Garofano L., Zhang J., Shi P., Lewis G., Sanson H. (2018). A metabolic function of FGFR3-TACC3 gene fusions in cancer. Nature.

[17] Bamber D. (1975). The area above the ordinal dominance graph and the area below the receiver operating characteristic graph. J. Math. Psychol..

[18] Schneider K., Venn B., Mühlhaus T. (2020). TMEA: A Thermodynamically Motivated Framework for Functional Characterization of Biological Responses to System Acclimation. Entropy.

[19] Liberzon A., Subramanian A., Pinchback R., Thorvaldsdóttir H., Tamayo P., Mesirov J.P. (2011). Molecular signatures database (MSigDB) 3.0. Bioinformatics.

[20] Sales G., Romualdi C. (2011). parmigene: A parallel R package for mutual information estimation and gene network reconstruction. Bioinformatics.

[21] Colaprico A., Silva T.C., Olsen C., Garofano L., Cava C., Garolini D., Sabedot T.S., Malta T.M., Pagnotta S.M., Castiglioni I. (2015). TCGAbiolinks: An R/Bioconductor package for integrative analysis of TCGA data. Nucleic Acids Res..

[22] Love M.I., Huber W., Anders S. (2014). Moderated estimation of fold change and dispersion for RNA-seq data with DESeq2. Genome Biol..

[23] Geistlinger L., Csaba G., Santarelli M., Ramos M., Schiffer L., Turaga N., Law C., Davis S., Carey V., Morgan M. (2020). Toward a gold standard for benchmarking gene set enrichment analysis. Brief. Bioinform..

[24] Garofano L., Migliozzi S., Oh Y.T., D’Angelo F., Najac R.D., Ko A., Frangaj B., Caruso F.P., Yu K., Yuan J. (2021). Pathway-based classification of glioblastoma uncovers a mitochondrial subtype with therapeutic vulnerabilities. Nat. Cancer.

[25] Huang D.W., Sherman B.T., Lempicki R.A. (2008). Bioinformatics enrichment tools: Paths toward the comprehensive functional analysis of large gene lists. Nucleic Acids Res..

[26] Bender E. (2016). Challenges: Crowdsourced solutions. Nature.

[27] Lim W.K., Lyashenko E., Califano A. Master Regulators Used As Breast Cancer Metastasis Classifier. Proceedings of the Pacific Symposium on Biocomputing.

[28] Chanda P., Costa E., Hu J., Sukumar S., Van Hemert J., Walia R. (2020). Information Theory in Computational Biology: Where We Stand Today. Entropy.

[29] Sarkar S., Hubbard J.B., Halter M., Plant A.L. (2021). Information Thermodynamics and Reducibility of Large Gene Networks. Entropy.

